# Towards specific inhibition of mTORC2

**DOI:** 10.18632/aging.101346

**Published:** 2017-12-12

**Authors:** Elizabeth R. Murray, Angus JM. Cameron

**Affiliations:** Kinase Biology Laboratory, Centre for Tumour Biology, Barts Cancer Institute, Queen Mary, University of London, John Vane Science Centre, Charterhouse Square, London, EC1M 6BQ, UK

**Keywords:** mTOR, mTORC2, AGC kinases, AKT, PKC, Sin1

The mammalian target of rapamycin (mTOR) serine/threonine protein kinase is a key regulator of eukaryotic cell growth, metabolism and survival. In mammals, mTOR functions in two distinct multi-subunit complexes, mTOR complex 1 (mTORC1) and mTOR complex 2 (mTORC2). These complexes integrate signals concerning the availability of cellular energy, nutrients and growth factors to affect metabolism, protein biosynthesis, lipid biosynthesis, cell proliferation and autophagy through the phosphorylation of distinct effectors, including members of the AGC kinase family. The target specificity of mTORC1 and mTORC2 can be attributed to the unique protein components of each complex. The mTORC1-specific subunit Raptor thus recruits mTORC1 substrates including 4EBP1 and p70S6 kinase. Amongst the various mTORC2-specific subunits, Sin1 has now been shown to recruit selected AGC kinase substrates including AKT. Both complexes are implicated in the aging process and multiple age-related diseases including cancer, cardiovascular disease, type II diabetes and neurodegenerative disorders.

Historically, investigation of mTOR complex function in aging and disease has been facilitated by genetic deletion of mTOR components, the small molecule inhibitor rapamycin, and ATP-competitive kinase inhibitors. Rapamycin is an acute inhibitor of mTORC1 but not mTORC2, and kinase inhibitors generally target both mTORC1 and mTORC2. Hence, whilst the role of mTORC1 in aging is relatively well defined – rapamycin is well established to extend the lifespan of mice – the study of mTORC2 function has been limited by a lack of specific mTORC2 inhibitors. This is complicated by the fact that chronic rapamycin treatment disrupts mTORC2 in a cell-type and context specific manner. For example, chronic rapamycin disrupts hepatic mTORC2 in vivo, leading to glucose intolerance and insulin resistance; attributed to mTORC2 as Rictor deletion alone also induces hepatic insulin resistance [1]. Intriguingly, Rictor deletion in mice is also deleterious for the longevity of males but does not negatively affect the lifespan of females, in a manner that is independent of glucose intolerance [2]. In a separate study, rapamycin treatment has been shown to increase the lifespan of both male and female mice, but to increase the lifespan of females more than males [3].

This suggests that chronic rapamycin-induced inhibition of mTORC2 limits the longevity benefits afforded by mTORC1 inhibition, specifically in male mice. Overall, these studies suggest caution in the use of non-specific mTOR complex inhibitors until more is understood with respect to the role of mTORC2 in aging and disease. Potential liabilities associated with targeting mTORC2, such as induction of diabetes and suppression of life-span, require further study.

**Figure 1:**
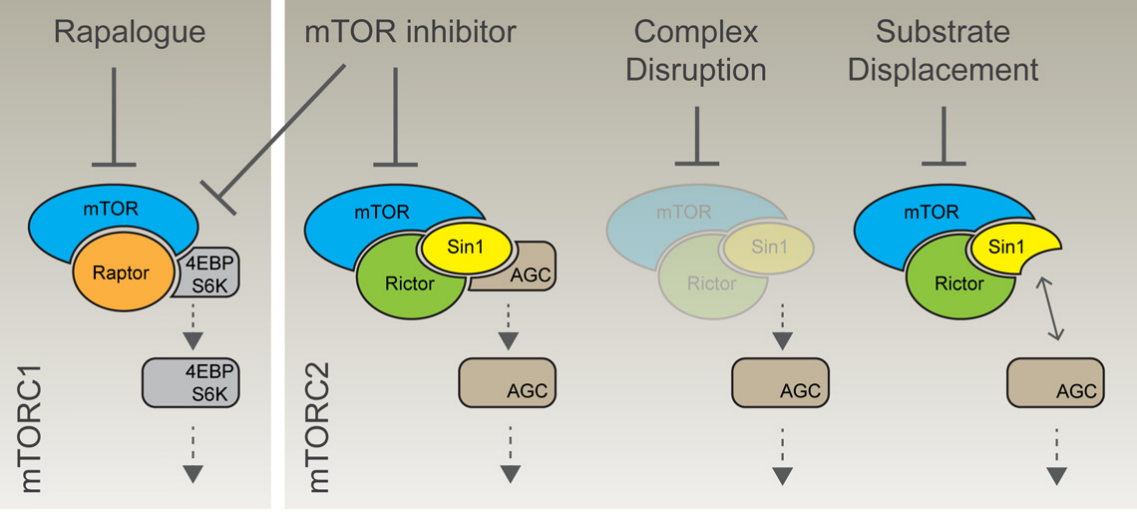
Distinct strategies for selective and unselective targeting of the mTOR complexes Rapamycin and related rapalogues acutely target mTORC1 specifically, although chronic exposure can also limit mTORC2 function in a context specific manner. Alternatively, mTOR catalytic inhibitors do not discriminate between the complexes. Targeting complex-specific subunits provides an alternative route to specific blockade. mTORC2 complex disruption, either through genetic deletion of Rictor or with small molecules, leads to loss of the entire complex. Alternatively, disruption of substrate recruitment can uncouple mTORC2 from its targets, while leaving the complex intact. Contrasting these distinct approaches will help define therapeutic opportunities and liabilities associated with mTOR inhibition.

Excitingly, new mechanisms of selectively inhibiting mTORC2 are emerging (Figure 1). One approach via disruption of mTORC2 substrate recruitment shows promise. We previously identified the central highly conserved region in the middle (CRIM) domain of Sin1 as a direct binding partner of the mTORC2-specific substrates PKCε and AKT [4]. Expression of Sin1 mutants, where the CRIM domain has been disrupted or deleted, is sufficient to displace endogenous Sin1 from mTORC2 and inhibit phosphorylation of the mTORC2-specifc targets AKT, PKCɑ and PKCε. In contrast, phosphorylation of the mTORC1-specific target, P70S6K Thr389 is unaffected. Recently, in a key paper by Tatebe et al., the CRIM domain of Sin1 was shown to form a discrete ubiquitin-fold domain that specifically binds and recruits mTORC2-specific substrate AGC kinases [5]. Target recognition requires interaction with a short acidic peptide, highly conserved in diverse eukaryote Sin1 orthologues, and previously shown by us to be required for AGC targeting [4]. In our most recent work [6], we have shown that expression of mutant Sin1 in DLD1 colon cancer cells inhibits AKT phosphorylation of the mTORC2 target site Ser473 and also on the PDK1 site Thr308. Furthermore, blocking mTORC2 substrate binding in DLD1 cells attenuated tumour growth, and tumour size correlated with the degree of AKT suppression in vivo. Thus, disrupting substrate recruitment provides a novel way to define mTORC2 complex-specific functions, and inhibitors of mTORC2-substrate interactions could be of therapeutic benefit for patients with cancer.

The next challenge is to identify feasible therapeutic strategies to selectively block mTORC2 function in the clinic. One possible approach is to target complex-specific protein-protein interactions, as pioneered recently with the identification of small molecules that inhibit the association of Rictor with mTOR [7]. These compounds inhibit the phosphorylation of mTORC2 targets AKT, NDRG1 and PKCα and critically, no effect on the phosphorylation of the mTORC1 substrate p70S6 kinase or mTORC1-dependent negative feedback loops was observed. In the same study, these small molecules inhibited cell growth, motility and invasion in glioblastoma cells in vitro and, in vivo, inhibited glioblastoma xenograft tumour growth. It is perhaps worth noting that pharmacological disruption or genetic ablation of the mTORC2 complex may not be the functional equivalent of blocking substrate recruitment (Figure 1). Indeed, a central highly conserved multi-protein complex such as mTORC2 is likely to have additional non-catalytic functions. Targeting substrate recruitment, either through CRIM domain mutation or with drugs, may thus provide a more nuanced view on mTORC2 function.

mTORC2 has emerged as a promising drug target, particularly in cancer, but the complex roles of mTORC2 in the aging process and age-related disorders remain potential liabilities. Progress in defining the molecular mechanisms underlying mTORC2 function has begun to identify selective strategies for targeting mTORC2, which will complement the mTORC1 directed rapalogues, as we continue to unravel the convoluted mTOR-signalling network.
